# Supplementary material for: Fast methane diffusion at the interface of two clathrate structures
Source: Nat Commun. 2017 Oct 20;8:1076. doi: 10.1038/s41467-017-01167-2 (PMC5715113; doi:10.1038/s41467-017-01167-2)
Supplement: Supplementary file 1 — Supplementary InformationSupplementary Information [file 41467_2017_1167_MOESM1_ESM.pdf]

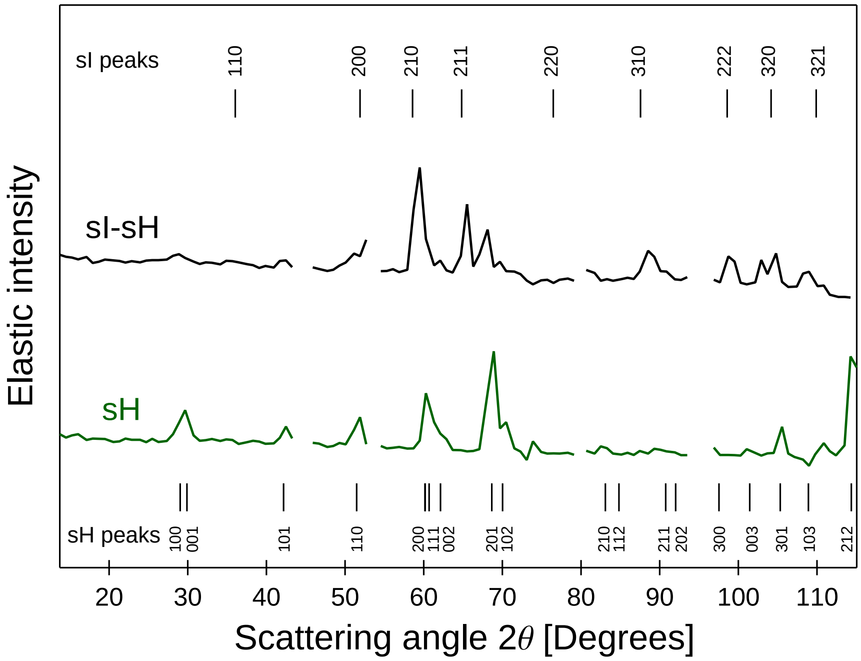

Supplementary Figure 1. Powder diffraction patterns of methane hydrate in pure clathrate sH at 1.4 GPa and 290 K and in the clathrate sI–sH at 1.0 GPa and 295 K. Breaks correspond to noisy detectors and to the strong Bragg peak of alumina in the anvils at 95°. The positions of the Bragg peaks for sI (cell parameter of 11.7 Å) and for sH (cell parameters of 11.7911 and 9.921 Å) are reported.

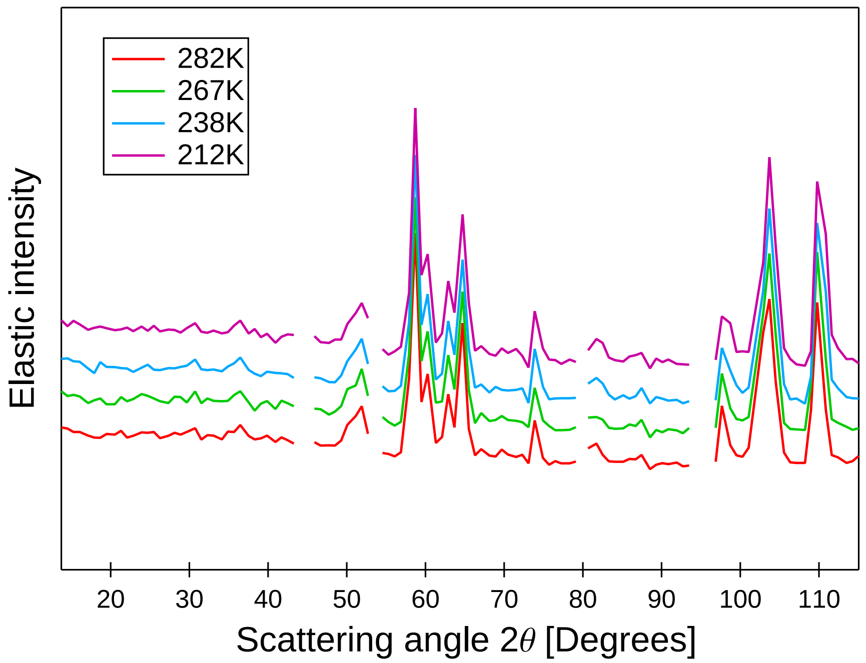

Supplementary Figure 2. Powder diffraction patterns of methane hydrate in the clathrate sI–sII at 0.8 GPa and the investigated temperatures. Breaks correspond to noisy detectors and to the strong Bragg peak of alumina in the anvils at 95°. The pattern at 282 K is also presented in Fig.1 of the main text.

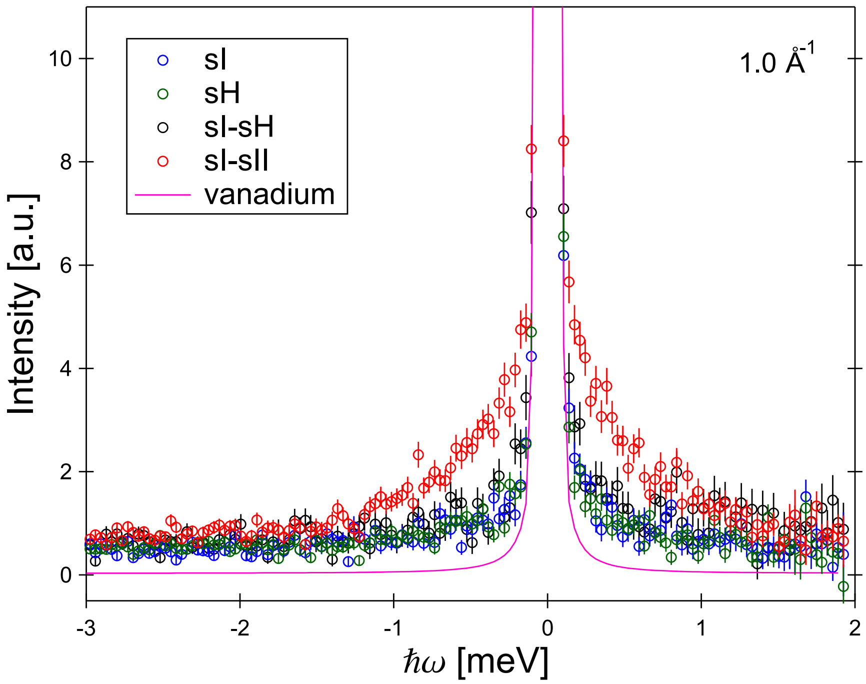

Supplementary Figure 3. Examples of QENS spectra of methane hydrate at 1.0 $Å^{-1}$ in pure structure I (at 0.4 GPa and 290 K), in pure structure H (1.4 GPa and 290 K), in the clathrate sI–sH (1.0 GPa and 295 K) and in the clathrate sI–sII (0.8 GPa and 282 K). Error bars were calculated by the square root of absolute neutron count combined with the law of propagation of errors. The instrumental resolution function is also shown.

Supplementary Note 1: Estimating the Population of Diffusing Extra-Cage Methane

We roughly estimated the population of methane molecules that is contributing to the quasielastic signal, based on the integrated areas of the quasielastic and elastic lines of the spectra. At 282 K, the ratio between the areas of the quasielastic and elastic components at 0.8 and 1.0 $Å^{-1}$, where no Bragg peaks are expected, is 0.4 with the 3D diffusion model and 0.45 with the 2D diffusion model. The relative temperature changes of those ratios are less than 10%.

The assumptions we used for this estimation are: i) the contribution from diffusing methane is completely comprised in the quasielastic component of the spectra, ii) the contribution from methane trapped in the clathrate is completely comprised in the elastic component of the spectra, iii) the contribution from methane is weighted by the total scattering cross section of CH_4, iv) the contribution from water is completely comprised in the elastic component of the spectra, v) the contribution from water is weighted by the total scattering cross section of D_2O and vi) there are 6 water molecules per methane molecule in the sample. Assumption iii does not consider that the rotational contribution to the spectra is in fact comprised in the flat background for methane of both populations; nevertheless, the rotational contribution is almost an order of magnitude less intense than the translational contribution at 0.8-1.0 $Å^{-1}$.

If we assume that for a cystalline matrix the D_2O coherent cross section does not contribute to the elastic component of the spectra, the result of the estimation is lowered by 15%. In the scenario of a partial decomposition of the water clathrate structure, the result of the estimation is lowered by 25% at most.

Supplementary Note 2: Estimating the Origin of the Diffusing Extra-Cage Methane

Let us first assume that no sI methane hydrate decomposes. In the starting sI clathrate hydrate cage occupancies are typically 86% for the small cages and 99% for the large cages. If similar occupancies are maintained in the sI hydrate and also characterise the sII hydrate of the sI-sII clathrate sample, then almost no methane can be released during transformation from sI to sII. However, if occupancies in sI are maintained but the cages of the sII hydrate contain a lower amount of methane, a significant fraction of methane could be released during the transformation. For example, one can calculate that approximately 10% of the methane in the sample is released if cage occupancies in sII are as low as 65% for the small cages and 85% for the large cages (based on the estimated composition of the sI-sII sample in terms of sI and sII, that is 2/3 and 1/3 respectively).

On the other hand, part of the diffusing extra-cage methane must originate from partial decomposition of the clathrate structure. Though the starting sI methane clathrate hydrate sample is in a stable and equilibrated phase, where all water molecules are part of the crystalline structure and all methane molecules are trapped in the cages of the structure, the compressed sample shows coexistence of stable structure I and metastable structure II and such coexistence in near equilibrium is likely characterised by a continuous dynamical rearrangement of water and methane molecules at phase boundaries. During the sI and sII coexistence, the two structures have been suggested to develop intercalated micrometer-sized thin layers[1] and disordered regions where methane is able to diffuse would form in between them. It must be noted that the liquid-like contribution of such

disordered regions to the diffraction patterns would be hardly detectable compared to a bulk amorphous or liquid. The previous estimation of a fraction of one third for the diffusing extra-cage methane suggests that a fraction of approximately 20-25% of the water molecules in the sample could belong to these disordered regions between clathrate sI and sII.

Supplementary Note 3: 2D Diffusion Model

For a particle restricted to move along a single plane, the scattering law is a Lorentzian whose half-width-half-maximum is $D_{2D}(Q)(Q\sin\theta)^2$, where $D_{2D}(Q)$ is the Q-dependent 2D translational diffusion coefficient and θ is the angle between the vector $\overrightarrow{Q}$ and the normal to the plane[2,3]. Then, for a polycrystalline sample where a large number of planes are oriented randomly the experimentally observed scattering law $< S_{2D}(Q,\omega) >_{orient.}$ is the isotropic orientational average:

$$< S_{2D}(Q,\omega) >_{orient.} = \frac{1}{2}\int_0^{\pi} \frac{1}{\pi}\frac{D_{2D}(Q)(Q\sin\theta)^2}{[D_{2D}(Q)(Q\sin\theta)^2]^2+\omega^2}\sin\theta \mathrm{d}\theta. \tag{1}$$

The integral can be calculated analytically and gives the following expression[2,3]:

$$< S_{2D}(Q,\omega) >_{orient.} = \frac{1}{8\pi k^3 D_{2D}(Q)Q^2}\times\left[\frac{1+k^2}{\cos\alpha/2}\ln\frac{1+2k\cos\alpha/2+k^2}{1-2k\cos\alpha/2+k^2}+\frac{2(1-k^2)}{\sin\alpha/2}\mathrm{arctg}\frac{2k\sin\alpha/2}{k^2-1}\right], \tag{2}$$

where

$$k = \left[1+\left(\frac{\omega}{D_{2D}(Q)Q^2}\right)^2\right]^{1/4},$$
$$\cos\alpha = \left[1+\left(\frac{\omega}{D_{2D}(Q)Q^2}\right)^2\right]^{-1/2},$$
$$\sin\alpha/2 = \left[\frac{1-\cos\alpha}{2}\right]^{1/2},$$
$$\cos\alpha/2 = \left[\frac{1+\cos\alpha}{2}\right]^{1/2}.$$

In our 2D diffusion data analysis, the expression for $< S_{2D}(Q,\omega) >_{orient.}$ given in equation (2) substituted the simple Lorentzian scattering law used in the 3D diffusion data analysis. Supplementary Fig. 4 depicts $\hbar D_{2D}(Q)Q^2$ as a function of Q^2 and is the 2D-analogous of Fig. 3 of the

main text. By analogy with the Singwi–Sjolander random jump diffusion model applied to normal 3D liquids, the coefficient $D_{2D}(Q)$ was fitted by $D_{2D}(Q) = D_{2D}/(1 + D_{2D}Q^2\tau_{2D})$, with D_{2D} the 2D translational diffusion coefficient and τ_{2D} the mean time between jumps[2].

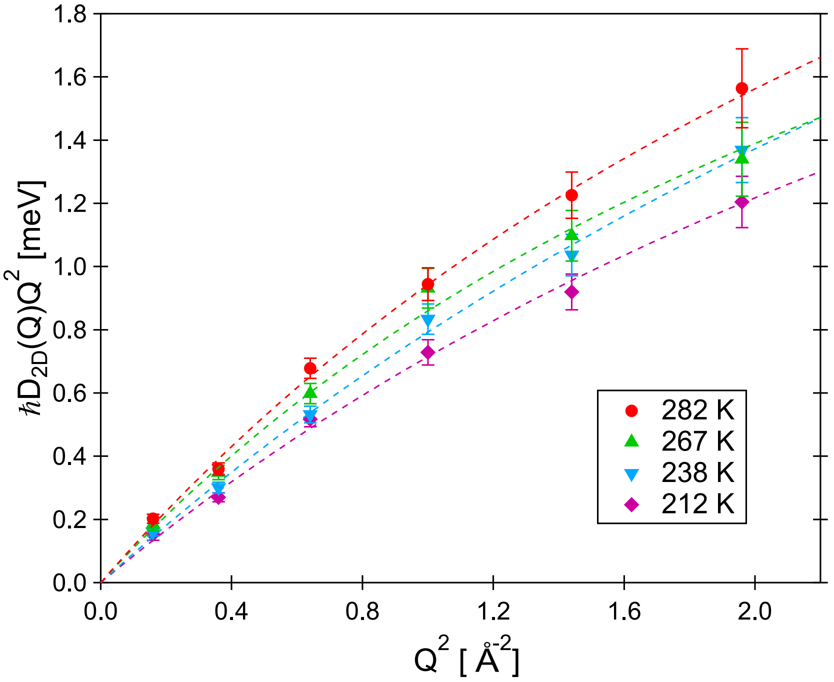

Supplementary Figure 4. $\hbar D_{2D}(Q)Q^2$ as a function of Q^2 at 0.8 GPa and the investigated temperatures as obtained from the 2D diffusion fits. Error bars correspond to one standard deviation. The best fits to the data according to a Singwi–Sjolander random jump diffusion model [$D_{2D}(Q) = D_{2D}/(1 + D_{2D}Q^2\tau_{2D})$] are shown as dashed lines. The values obtained for D_{2D} and τ_{2D} are reported in Fig. 4 of the main text.

Supplementary References

1. Schicks, J. M. & Ripmeester, J. A. The coexistence of two different methane hydrate phases under moderate pressure and temperature conditions: Kinetic versus thermodynamic products. *Angew. Chem., Int. Ed.* **43**, 3310–3313 (2004).

2. Dianoux, A. J., Volino, F. & Hervet, H. Incoherent scattering law for neutron quasi-elastic scattering in liquid crystals. *Mol. Phys.* **30**, 1181–1194 (1975).

3. Lechner, R. E. Effects of low-dimensionality in solid-state protonic conductors. *Solid State Ionics* **77**, 280–286 (1995).
